# Assessment of Dynamic Knee Valgus between Lateral Step-Down Test and Running in Female Runners with and without Patellofemoral Pain Using Two-Dimensional Video Analysis

**DOI:** 10.3390/clinpract12030047

**Published:** 2022-06-10

**Authors:** Diego Protasio de Vasconcelos, Felipe J. Aidar, Tarcisio Brandao Lima, Flavio Martins do Nascimento Filho, Igor Leonardo Alves Mendonça, Alfonso López Díaz-de-Durana, Nuno Domingos Garrido, Michael Silveira Santiago, Walderi Monteiro da Silva Junior

**Affiliations:** 1 Postgraduate Program of Physical Education, Federal University of Sergipe (UFS), São Cristovão 49100-000, Brazil; walderi@academico.ufs.br; 2 Musculoskeletal System Unit, University Hospital, Federal University of Sergipe (UFS), Aracaju 49060-025, Brazil; michael.santiago@ebserh.gov.br; 3 Postgraduate Program of Health Sciences, Federal University of Sergipe (UFS), São Cristovão 49100-000, Brazil; brandao_doutorado@academico.ufs.br (T.B.L.); flaviomartinsfilho@academico.ufs.br (F.M.d.N.F.); 4 Department of Physical Therapy, Federal University of Sergipe (UFS), São Cristovão 49100-000, Brazil; igor10leo@academico.ufs.br; 5 Sports Department, Physical Activity and Sports Faculty-INEF, Universidad Politécnica de Madrid, 28040 Madrid, Spain; alfonso.lopez@upm.es; 6 Research Center in Sports Sciences, Health Sciences and Human Development (CIDESD), University of Trás-os-Montes e Alto Douro, 5001-801 Vila Real, Portugal; ngarrido@utad.pt

**Keywords:** knee, running, women, patellofemoral pain syndrome, biomechanical phenomena

## Abstract

Dynamic knee valgus (DKV) is a frontal plane knee kinematic alteration that has been associated with patellofemoral pain (PFP) in female runners. DKV is commonly assessed in clinical practice by measuring frontal plane knee projection angle (FPPA) during squat tests. However, it remains unclear whether the DKV observed in these tests is similar to or correlates with that observed during running in female runners. The aims of this cross-sectional study were to correlate and compare DKV, by measuring FPPA values, in a lateral step-down (LSD) squat test and running in female runners with and without PFP. A two-dimensional (2D) video analysis of the LSD test and running was carried out for 21 asymptomatic female runners and 17 PFP female runners in order to determine FPPA values. A Pearson correlation test and a factorial ANOVA with Bonferroni post hoc correction were used for statistical analysis. The FPPAs recorded in the LSD test were significantly higher than those recorded during running in the asymptomatic (16.32° ± 5.38 vs. 4.02° ± 3.26, *p* < 0.01) and PFP groups (17.54° ± 7.25 vs. 4.64° ± 3.62, *p* < 0.01). No significant differences were found in FPPA values between asymptomatic and PFP runners during the LSD test (16.32° ± 5.38 vs. 17.54° ± 7.25, *p* = 0.55) and running (4.02° ± 3.26 vs. 4.64° ± 3.62, *p* = 0.58). There was a small (*r* < 0.3) and non-significant (*p* > 0.05) correlation in FPPAs between the LSD test and running in both groups. According to our results, DKV was not similar during the LSD test and running, and there was no significant correlation in FPPA values between the LSD test and running in both groups. Therefore, clinicians and therapists should be aware of these findings when using the LSD test in clinical practice to evaluate DKV in female runners with or without PFP.

## 1. Introduction

Patellofemoral pain (PFP) is among the most common causes of knee injury in recreational runners [1] and is responsible for 20 to 40% of knee pain related to sports practice [2]. It affects twice as many women as men [3] and may reduce quality of life and restrict participation in physical activities [4,5]. PFP is a clinical syndrome characterized by the presence of pain in the anterior region of the knee, around or behind the patella, occasionally accompanied by knee effusion or crepitus [4]. The symptoms are aggravated by activities that loads the patellofemoral joint, such as squatting, stair ambulation, running or jumping [4]. Its etiology is controversial and has been associated with biomechanical alterations of the hips (e.g., decreased strength of abduction and external rotation), knees (e.g., lower knee extension strength) and feet (e.g., rearfoot eversion and reduced dorsiflexion) [4,6,7]. These alterations contribute to the development of lower limb abnormal movement patterns during activities, such as dynamic knee valgus (DKV) [8,9,10]. Abnormal knee valgus is related to an increase in the contact pressure between the articular surfaces of the patella and the femoral trochlea [6], resulting in the onset of pain and secondary inflammatory chondral alterations [8].

DKV is an abnormal movement pattern characterized by increased adduction and internal rotation of the femur, knee abduction, external rotation of the tibia and eversion of the ankle during dynamic tasks, such as squatting, jump landings or running [11]. The inability to maintain adequate dynamic knee alignment during movements or physical exercises may lead to knee injuries [8]. Previous studies have shown that DKV is an important risk factor for PFP [9,12] and anterior cruciate ligament injury [13]. Since dynamic valgus is a corrigible risk factor [14], early detection in clinical practice and proper management are crucial to preventing lesions. For this purpose, lower limb functional tests have been used clinically to assess individuals at risk of lower limb injuries and detect altered movement patterns, such as DKV [15].

Squat tests are one of the most commonly used lower limb tests in clinical practice and represent a simple and accessible form of clinical movement assessment that can be performed outside the laboratory in a clinical or training setting [16,17]. The lateral step-down (LSD) test is one such established squat test, widely used to assess knee movement pattern [18]. It has been described as the most sensitive test to detect kinematic alterations in individuals with PFP [19]. DKV is commonly quantified during squat tests by measuring the frontal plane knee projection angle (FPPA) at peak knee flexion [20] using two-dimensional (2D) video analysis [17]. This is a reliable and reproducible measurement with comparable results to 3D analysis [21,22].

Some studies [23,24] have shown that individuals with PFP presented higher degrees of FPPA during squat tests compared to asymptomatic subjects. However, it remains unclear whether the FPPAs measured during squat tests are similar to or correlate with those measured during running. The relationship between knee valgus and PFP in running is controversial, with some authors supporting this link [25,26] and others contesting it [27,28]. Only two previous studies compared or correlated the frontal plane kinematics between squat tests and running. Rees et al. [27] found no correlation in FPPA between the single-leg squat test and running in runners with PFP, whilst Alenezi et al. [29] found a correlation between the single-leg squat test and running in recreational athletes. In these two studies, the samples were heterogeneously composed of males and females and the analyzed test was the single-leg squat test, which presents kinematic differences to the LSD test [19]. To our knowledge, no study to date has compared or correlated DKV in LSD tests and running in female runners with and without PFP. The assumption that movement patterns detected in the LSD test would correlate with those during running has no sufficient evidence [27]. Since the LSD test is widely used in clinical practice by clinicians and therapists to evaluate DKV [18,19,30], this lack of evidence should be addressed in order to support the assessment of DKV in female runners using this test.

Our study aimed to correlate and compare DKV, by measuring FPPA, between the LSD test and running in female runners with PFP and asymptomatic female runners. Our main hypotheses were (1) that FPPA values in the LSD test would correlate with FPPA in running and (2) that FPPA values in the LSD test would be similar to FPPA values in running, in both groups. Our secondary hypothesis was that PFP female runners would present higher FPPA values than asymptomatic female runners during both the LSD test and running.

## 2. Materials and Methods

### 2.1. Participants and Design

A cross-sectional observational study was carried out after approval by the Research Ethics Committee of our institution (CAAE 43040721.2.0000.5546) and was conducted according to the guidelines of the Declaration of Helsinki. The volunteers were informed about the details of the research and those who agreed to participate signed an informed consent form. Sample size was defined by a priori sample calculation using G*Power software (G*Power 3.1.9.6, Kiel University, Kiel, Germany) based on a statistical power of 80% (β = 0.2), a significance level of 5% (α = 0.05) and FPPA mean values and standard deviations during squat tests (control: 8.4° ± 5.1; PFP: 16.8° ± 5.4) and running (control: 13.5° ± 5.7; PFP: 21.7° ± 3.6), according to a previous study [23]. The final sample consisted of 38 female runners, 21 asymptomatic and 17 with PFP.

**Figure 1:**
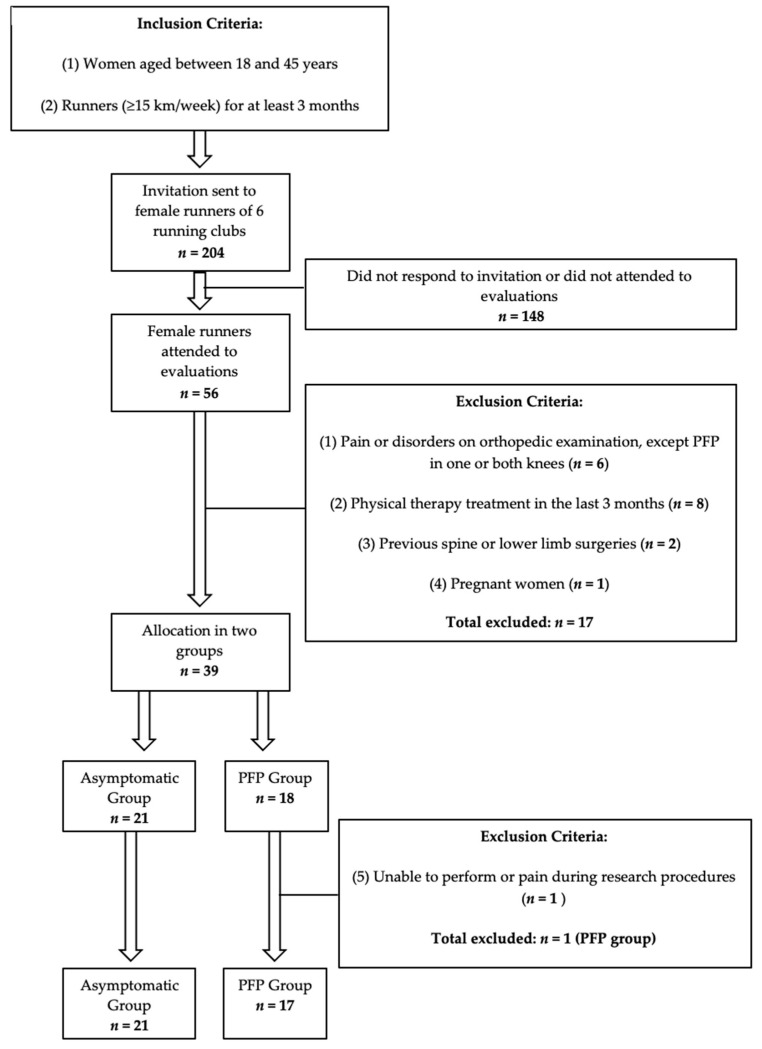
Participant selection process chart. Abbreviations: PFP: patellofemoral pain.

Participants were recruited from six running clubs located in the city of Aracaju, Brazil. All female runners in these clubs (*n* = 204) were invited by email and text message to attend a research evaluation if they met the inclusion criteria: aged 18–45 years and had been running regularly, at least 15 km/week, for at least 3 months [25,31,32] (Figure 1). This age group was defined to avoid skeletally immature individuals and individuals with knee osteoarthritis (OA), which usually affects females over age 45 [4]. A total of 56 female runners responded to the invitation and attended evaluations. The exclusion criteria were: causes of pain or musculoskeletal disorders, except PFP in one or both knees; previous surgeries on the spine, pelvis or lower limbs; physical therapy treatment in the last 3 months; pregnancy; previous history of joint infection or inflammatory diseases; and inability to perform or experience of pain during research procedures (Figure 1).

In order to identify the exclusion criteria, all participants underwent a complete orthopedic clinical examination performed by an experienced orthopedic surgeon specialized in knee disorders. PFP diagnosis is clinical, based on clinical history and physical examination, and does not require imaging tests [4,12]. For the PFP diagnosis, the orthopedic surgeon used the consensus criteria for running-related injuries proposed by Yamato et al. [33]. According to this consensus, PFP diagnosis is defined by the presence of insidious anterior knee pain related to training or running with onset of symptoms for at least 3 months, reported average pain in the last 3 months with a minimum intensity of 3 (0–10) on the Visual Analog Pain Scale (VAS) and pain reproduced by at least one of the following situations: squatting, kneeling, sitting for long periods and going up or down stairs. During clinical examination, specific retropatellar or peripatellar pain on palpation, anterior knee pain on squatting and positive provocative patellofemoral tests (e.g., patellar compression, anterior pain on resisted quadriceps contraction) were used to further increase the diagnostic accuracy of PFP [4,34]. Participants that presented any others causes of knee pain or tenderness, alterations on physical examination or suspected knee OA were excluded. A total of seventeen (*n* = 17) runners were excluded and the remaining thirty-nine participants were allocated to the asymptomatic group (*n* = 21) or the PFP group (*n* = 18) (Figure 1). During procedures, one participant (*n* = 1) in the PFP group experienced pain during running and was excluded.

After allocation, the participants were identified by a badge with a number obtained by a randomization tool (www.random.org, accessed on 28 August 2021) that was adjusted to generate a non-repeated random number (from 0 to 100) for each participant by a researcher not involved with the procedures and video analysis phases. The researchers involved in the procedures and video recording, as well as the researcher who measured FPPAs using these videos, did not have access to randomization. They were blinded in relation to the group and the symptomatic limb of the participants and registered only the badge number on the evaluation form for each participant. Thus, allocation concealment was preserved.

### 2.2. Procedures

All participants underwent the following procedures: anthropometric assessment, running and the LSD test. All procedures were performed on the same day for each participant, who wore usual, comfortable sportswear that did not restrict their movements, as well as their own usual running shoes and socks [34]. Everyone warmed up on a stationary bicycle for 5 min [25] at a comfortable speed and then performed the LSD test and running, in that order, with a 2 min resting break between the LSD test and running. Participants who failed to perform or who experienced pain during the LSD test or running had to end the procedure and were excluded.

#### 2.2.1. Lateral Step Down (LSD)

Each volunteer performed, after verbal and video explanation, five sequential LSD repetitions, the first one for familiarization and the others valid. The test consisted in performing a unipodal squat from the initial position in which the evaluated lower limb rested on the edge of the platform (20 cm high) while the non-evaluated limb was pending laterally to it, with full knee extension and maximum ankle dorsiflexion [9]. The unipodal squat was performed until the heel of the suspended limb reached the ground, at which point the participant returned to the home position [9,19]. Approximately 60 degrees of knee flexion was desired, controlled by a goniometer, for which wooden blocks were used, both on the ground and under the limb supported on the platform, in order to adjust the test execution to participant height [9,19]. The tests lasted approximately 5 s, measured by a stopwatch, with 2 s for descent, 1 s for heel touch and 2 s for ascent to the starting position [19]. In cases of trunk or limb imbalance or incorrect execution, the tests were repeated.

#### 2.2.2. Running

The running was performed on an ergometric treadmill model LX Classic 3.0 (Movement^®^, São Paulo, Brazil) with a running area of 42 cm. The volunteers were instructed to start the run at a low speed and, gradually and slowly, to increase it until reaching their usual pace, after which they were to maintain this pace for 5 min [27]. This technique has proven effective in evaluating runners when compared to the use of predetermined speeds [35]. After the fifth minute of running at their usual pace [27], 10 s of running were recorded on video and after that the volunteers were instructed to stop running.

### 2.3. Analysis

#### 2.3.1. Data Processing and Reduction

**Figure 2:**
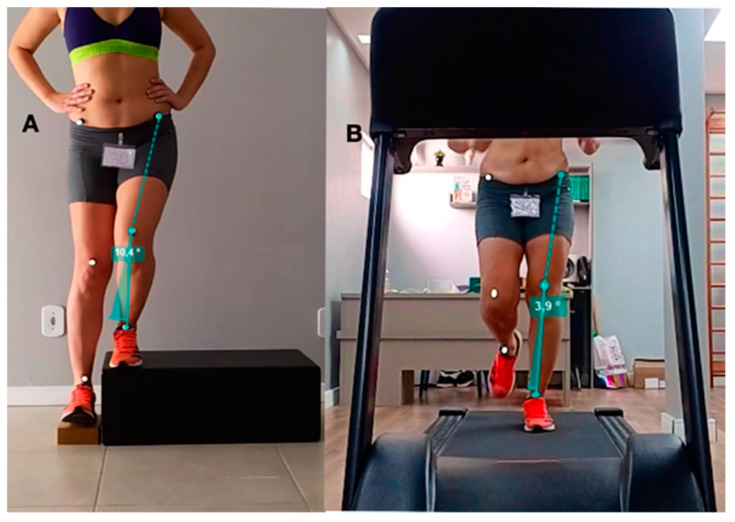
Frontal plane knee projection angle (FPPA) measured during lateral step down (**A**) and running (**B**).

The data recorded in the anthropometric assessment were weight (kg), height (cm) and body mass index (BMI = weight kg/height cm^2^). During running and the LSD test, FPPA for both lower limbs was measured and recorded by the angle formed by a line drawn from the anterosuperior iliac spine (ASIS) to the center of the knee and another line drawn from the center of the knee to the center of the ankle [23,27]. FPPA is a reliable and reproducible 2D measurement of frontal plane lower limb kinematics [21,22] with comparable results to 3D analysis [21]. Self-adhesive reflective markers were used on the participants’ skin at the three body landmarks (ASIS, the center of the knee at mid-distance between the medial and lateral femoral condyles, and the center of the ankle) for FPPA measurement (Figure 2).

The running and LSD test were video-recorded at 240 frames per second (fps) in high-definition (1080p) resolution using a digital camera (Iphone 12, Apple Inc., Cupertino, CA, USA) attached to a static tripod and positioned at a distance of 3 m and at a height equivalent to the participant’s knee level [21]. The recorded videos were viewed frame by frame using the open software Kinovea (Version 0.9.5) [36,37] in order to obtain the moment of FPPA measurement in both limbs, which was measured and recorded by a single researcher. This FPPA measurement moment was the peak of knee flexion which in running occurred during unipodal support, with the foot fully supported and the patella reaching the most caudal projection during knee flexion [21,22,27] (Figure 2). During LSD, this moment was when the heel of the contralateral limb touched the ground or the lateral wooden block [22] (Figure 2). For the LSD test, the arithmetic mean of the FPPAs measured in 4 valid repetitions in each lower limb of the participants was recorded. For running, the arithmetic mean of two measurements of FPPA in each limb was recorded. For data reduction purposes, we considered for analysis in the PFP group only FPPA values measured in the limb diagnosed with PFP or in the most painful one according to the VAS, when bilateral. In the asymptomatic group, the analysis considered PFPA values measured in the dominant lower limb.

#### 2.3.2. Statical Analysis

The numerical variables were submitted to the Shapiro–Wilk normality test, which indicated a parametric data distribution (*p* > 0.05), and were presented as means and standard deviations (SDs). The demographic and anthropometric data were compared between the groups using an independent *t*-test in order to demonstrate that there was no difference between the groups regarding these variables. Levene’s test showed that the variances of the groups in the LSD test and running were equal (SDL: F (1.36) = 2.329, *p* = 0.136) (Running: F (1.36) = 0.009, *p* = 0.926). A factorial ANOVA with Bonferroni post hoc correction was conducted to compare FPPAs between the two situations, the LSD test and running, and the two groups. A Pearson correlation test was used to assess correlation in FPPA values between running and the LSD test in the two groups. The strength of the correlation was described using the guidelines proposed by Hopkins et al. [38]: trivial (*r* = 0.0 to 0.1), small (*r* = 0.1 to 0.3), moderate (*r* = 0.3 to 0.5), large (*r* = 0.5 to 0.7), very large (*r* = 0.7 to 0.9) and extremely large (*r* = 0.9 to 1.0). Statistical analysis was performed using IBM SPSS^®^ (*Statistical Package for Social Sciences*, version 22.0) software and statistical significance was defined as *p* < 0.05.

## 3. Results

The groups were similar for age, weight, height, BMI, running time, weekly training distance and running speed (Table 1). The FPPAs measured during LSD were significantly higher than those measured during running in the asymptomatic (16.32° ± 5.38 vs. 4.02° ± 3.26, *p* < 0.01) and PFP groups (17.54° ± 7.25 vs. 4.64° ± 3.62, *p* < 0.01) (Table 2). There was a small correlation without statistical significance between the FPPA values measured during the LSD test and running in the asymptomatic group (*r* = 0.16, *p* = 0.46) and the PFP group (*r* = 0.27, *p* = 0.28) (Table 2). When comparing asymptomatic and PFP female runners, no statistically significant differences were found in FPPA values during LSD (16.32° ± 5.38 vs. 17.54° ± 7.25, *p* = 0.55) and running (4.02° ± 3.26 vs. 4.64° ± 3.62, *p* = 0.58) (Table 3).

## 4. Discussion

In our research, we studied frontal plane knee kinematics using 2D video analysis of female runners with patellofemoral pain and asymptomatic female runners in order to correlate and compare DKV, by measuring FPPA, in two different situations: during the LSD test and running. Although there are previous studies [27,29] that have compared FPPA during squat tests and running, we did not identify a study that compared it between the LSD test and running in female runners. Our hypotheses were that the FPPA values measured in the LSD test would be similar and correlate with those measured in running, in both groups, and that female runners with patellofemoral pain would have higher FPPA values in the LSD test and running when compared to asymptomatic female runners. However, our results were different from what we had hypothesized. We found no correlation in FPPA measurements between the LSD test and running, and FPPA measured in the LSD test was significantly higher than in running, in both groups. Furthermore, there was no significant difference in FPPA between groups during the LSD test and running.

Pompeo et al. [39] found no differences in FPPA measured during the forward step-down squat test between women with PFP and women who were asymptomatic. Although similar to our results, this test presents some kinematic differences in relation to the LSD test due to the positioning of the contralateral limb. In the forward step-down test, the contralateral limb is positioned pending forward, while in the LSD it is positioned laterally to the platform during squatting. This difference means a demand for more muscle recruitment to control frontal plane alignment during the LSD test and could explain the finding that the LSD test was more sensitive than the forward step-down test in the detection of DKV in PFP and asymptomatic women [19]. On the other hand, other authors [23,29,39] have reported that women with PFP presented higher FPPA in single-leg squat tests when compared to asymptomatic women. In our study, we found no difference in DKV between the asymptomatic and PFP groups during the LSD test. These differences in the literature could be explained, according to Powers CM et al. [7], by the fact that kinematic changes would not always be present in all individuals with PFP as well as the fact that there may be an asymmetry in Q angles between individuals [40].

We found only two previous studies comparing or correlating DKV between squat tests and running. In a study involving 15 young adults of both genders who were recreational athletes, Alenezi et al. [29] found a correlation between the single-leg squat test and running. Rees et al. [27] compared and correlated FPPA values between the single-leg squat test and running in 16 PFP runners and 16 asymptomatic runners of both genders. They found no correlation between the single-leg squat test and running in PFP runners. Conversely, they identified a significant correlation in FPPA between single-leg squats and running in asymptomatic runners [27]. When comparing the FPPA values during running, these authors [27] did not find significant differences between the groups. However, during the single-leg squat test, PFP runners had higher FPPA values than asymptomatic runners [27]. These findings are partially in agreement with ours. In our sample, we found no significant correlation between LSD and running in PFP female runners and asymptomatic runners. We also found no difference in FPPA values between the groups, both during the LSD test and running. These differences could be due to some kinematic discrepancies between the single-leg squat and LSD tests [19]; moreover, our sample consisted only of female runners while the other studies included individuals of both genders.

Noehren B et al. [28], in a 3D kinematic analysis, also found no differences in DKV measured during running between asymptomatic and PFP women, even during thirty-minute prolonged running that caused onset of knee pain in the PFP group. Otherwise, Dierks et al. [25], also in a 3D analysis, found higher DKV values in individuals with PFP compared to asymptomatic individuals during prolonged running causing fatigue. In a recent study, Fidai MS et al. [41] demonstrated that, after the application of a fatigue protocol in young athletes, there was an increase in DKV during the drop jump test. Although conflicting, these findings in the literature suggest that the kinematic changes could be influenced by fatigue and are likely to occur after a certain amount of running time. In our study, as in a previous study [29], the running protocol consisted of running on a treadmill at the usual pace [35] for 5 min and video was recorded for 10 s after this time. It may be that the running time was too short to trigger possible kinematic changes related to fatigue, such as DKV, which could explain the non-correlation between FPPA values measured in the LSD test and running in the groups included in our sample. We suggest that future studies should evaluate running protocols of different durations and compare the results in order to investigate whether running time and fatigue influence DKV during running in asymptomatic and PFP female runners. In our study, participants were excluded if they experienced pain during running or the LSD test, which occurred with only one participant in the PFP group. Using these criteria, we sought to avoid the effect of pain on triggering abnormal movements in both groups. We recommend that future research assess the relationship between the onset of pain and the immediate kinematic changes in female runners.

Our study has some limitations. Although our sample size met a priori sample calculation criteria and was similar to previous studies, larger samples could help to clarify the comparison and correlation in FPPA values between the LSD test and running. As previously described, there may be an asymmetry in Q angles between individuals [40] and it may be that not all individuals with PFP would present kinematic changes [7]. The absence of different running times and fatigue protocols is another limitation of our study. Even though the matter is controversial [25,28] fatigue could trigger abnormal movements in running, and this relationship needs to be evaluated in female runners in further research. In our research, we decided that the participants should run at their usual paces during running, the decision being based on a previous study [35] which concluded that a predetermined running speed was not relevant to DKV values during running. However, this study analyzed asymptomatic individuals of both genders, so we cannot assert that these results could be extrapolated to female runners with PFP. This is a limitation, and we suggest that future research should analyze whether running speed influences frontal plane kinematics by measuring FPPA values at different running speeds in female runners with PFP.

DKV is a well-known modifiable risk factor for patellofemoral pain syndrome [9,12,14,42]—a running-related injury [4,43,44]. Reliable evaluation and earlier detection of DKV is important to define interventions with the capacity to reduce FPPA in order to prevent or manage lesions [15,44]. There is a need for a relatively low-cost assessment to detect DKV in clinical practice using functional tests that can reproduce abnormal movements that would occur during running [8]. The LSD test has been recommended and widely used by clinicians, physiotherapists and orthopedic surgeons to evaluate dynamic valgus in PFP patients [18,19,30]. Nevertheless, there is a lack of evidence regarding whether the DKV observed during squat tests is correlated with or similar to that observed in running [27]. To our knowledge, our study is the first to compare and correlate the DKV between the LSD test and running in female runners with and without PFP. Our findings showed that FPPA values were neither correlated nor similar between the LSD test and running in PFP female runners and asymptomatic female runners. Thus, we recommend that clinicians and therapists be aware of our findings when using the LSD test to evaluate DKV in female runners with and without PFP.

## 5. Conclusions

Our study found no correlation in DKV, by measuring FPPA, between the LSD test and running in female runners with PFP and asymptomatic female runners. The FPPAs measured in the LSD test were significantly higher than those measured during running in both groups. When comparing asymptomatic female runners and PFP female runners, no statistically significant differences were found in FPPA values during the LSD test and running. Thus, in our sample, the LSD test did not reproduce the DKV observed during running in both groups. We conclude that the LSD test should be used with caution in clinical practice to predict DKV in running in asymptomatic and PFP female runners.

## Figures and Tables

**Table 1:** Means and standard deviations (SDs) of age, anthropometric data, running time and weekly training distance in asymptomatic and PFP groups.

Variables	Group		*p*	CI 95%
	Asymptomatic (*n* = 21)	PFP (*n* = 17)		
Age (years)	35.81 ± 6.32	36.00 ± 7.55	0.933	(−4.37; 4.75)
Weight (kg)	61.91 ± 7.10	65.21 ± 11.11	0.274	(−2.72; 9.32)
Height (cm)	163.23 ± 6.36	163.11 ± 7.40	0.957	(−4.65; 4.40)
BMI (kg/cm^2^)	23.23 ± 2.21	24.48 ± 3.73	0.207	(−0.72; 3.22)
Running time (months)	40.81 ± 30.40	56.00 ± 58.78	0.311	(−14.76; 45.14)
Weekly training distance (km/week)	21.19 ± 6.18	20.59 ± 5.73	0.760	(−4.56; 3.36)
Running speed (m/s)	2.42 ± 0.29	2.38 ± 0.36	0.707	(−0.17; 0.25)

Abbreviations: PFP: patellofemoral pain; CI: confidence interval (at 95%); BMI: body mass index.

**Table 2:** Means and standard deviations (SDs) of the frontal plane knee projection angle (FPPA). Comparison and correlation between the LSD test and running in the two groups. Eta-squared (*η*^2^) and Pearson’s correlation coefficient (*r*).

Group	FPPA (°)		*p*	*η* ^2^	CI 95%	*r*	*p*
	LSD	Running					
Asymptomatic	16.32 ± 5.38 *	4.02 ± 3.26	<0.01 *	0.680	(9.44; 15.15)	0.16	0.465
PFP	17.54 ± 7.25 #	4.64 ± 3.62	<0.01 #	0.654	(9.73; 16.07)	0.27	0.289

* Indicates a statistically significant difference from running in the asymptomatic group. # Indicates a statistically significant difference from running in the PFP group. No statistically significant correlation between LSD and Running for the Asymptomatic and PFP groups. Abbreviations: PFP: patellofemoral pain; CI: confidence interval (at 95%).

**Table 3:** Means and standard deviations (SDs) of the frontal plane knee projection angle (FPPA) during lateral step down (LSD) and running. Comparison between groups.

FPPA (°)	Group		*p*	*η* ^2^	CI 95%
	Asymptomatic	PFP			
LSD	16.32 ± 5.38	17.54 ± 7.25	0.555	0.010	(−5.38; 2.93)
Running	4.02 ± 3.26	4.64 ± 3.62	0.588	0.008	(−2.88; 1.65)

No statistically significant difference between the Asymptomatic and PFP groups from LSD and running. Abbreviations: PFP: patellofemoral pain; Eta-squared (*η*^2^); CI: confidence interval (at 95%).

## Data Availability

The data presented in this study are available upon request from the corresponding author.
